# Study on the Therapeutic Effects of Drug and Cognitive-Behavioral Therapy on Non-Erosive Reflux Disease Patients With Emotional Disorders

**DOI:** 10.3389/fpsyt.2018.00115

**Published:** 2018-05-09

**Authors:** Xiuhua Li, Fengjiao Ding, Pandeng Luo, Jing Yang, Zhenhua Liu, Jinwei Liu, Yali Zhang, Aimin Leng, Kuangming Wu

**Affiliations:** ^1^Department of Gastroenterology, Yiyang Central Hospital, Yiyang, China; ^2^Yiyang No.1 Middle School, Yiyang, China; ^3^Department of Gastroenterology, The First Affiliated Hospital of University of South China, Hengyang, China; ^4^Department of Nephrology, Yiyang Central Hospital, Yiyang, China; ^5^Department of Intensive Care Unit, Yiyang Central Hospital, Yiyang, China; ^6^Department of Gastroenterology, Xiangya Hospital, Central South University, Changsha, China

**Keywords:** non-erosive reflux disease, cognitive-behavioral therapy, drug therapy, anxiety, depression

## Abstract

**Objective:**

To assess the correlation between the incidence of non-erosive reflux disease (NERD) and mental and psychological factors, deepen the understanding of the pathogenesis of NERD and explore effective treatments.

**Methods:**

NERD patients with mood disorders who met the inclusion criteria were randomly divided into a drug treatment group, a psychotherapy group, and a psychotherapy combined with drug treatment group. Before and after treatment, the patients were retrospectively analyzed using the gastroesophageal reflux disease Questionnaire, Hamilton Depression Scale, Hamilton Anxiety Scale, and SF-36 Quality of Life Scale.

**Results:**

All three treatments were found to relieve patients’ symptoms and improve their quality of life to some extent. The psychotherapy combined with drug treatment group showed the best overall curative effect. The Hamilton Depression and Anxiety Scale scores were significantly lower in the psychotherapy-alone group and psychotherapy combined with drug treatment group than in the drug treatment alone group at 4, 8, and 12 weeks (*P* < 0.05).

**Conclusion:**

Medication, psychotherapy, and psychotherapy combined with medication can relieve clinical symptoms and improve quality of life to varying degrees in patients with NERD.

## Introduction

Gastroesophageal reflux disease (GERD) is a disease in which the gastroduodenal contents reflux into the esophagus, causing such symptoms and complications as acid reflux, heartburn, and post-sternal pain (1). GERD includes non-erosive reflux disease (NERD), reflux esophagitis (RE), and Barrett’s esophagus (2, 3). NERD, the most common form of GERD, has the same symptoms as other forms but does not involve endoscopic esophageal mucosal damage or erosion (4). NERD accounts for about 70% of GERD (2). The spectrum of NERD symptoms is rather complex. NERD involves heartburn, acid reflux, and other typical symptoms of reflux, but it is also often associated with typical non-esophageal symptoms, such as chronic cough, pharyngitis, precordial pain, and asthma (5), which seriously affect the patients’ quality of life (6, 7).

Studies have shown that the incidence of NERD has been increasing annually (8, 9), especially in middle-aged and elderly women. Research shows that most patients with NERD often also suffer from anxiety, depression, or other emotional disorders (10–12). At present, the etiology and pathogenesis of NERD remain unclear. The current studies suggest that the pathogenesis of NERD may be related to visceral hypersensitivity (13, 14), esophageal motility, esophageal acid exposure, and esophageal mucosal barrier changes. Meanwhile, it is found that the severity of NERD is related to social and psychological stress and has a clear relationship with mental factors, such as anxiety and depression (15). Currently NERD treatment has no uniform standard. There is no one drug suitable for all patients with NERD nor is there any drug that absolutely cures the condition. Conventional NERD treatment options include acid suppression, gastrointestinal motility drugs, and mucosal protective agents. Proton-pump inhibitors (PPIs) are currently the most commonly used drug for the treatment of NERD (16). Studies have found that NERD patients respond less well to various PPIs than RE (17) and NERD patients with the use of conventional PPI and gastrointestinal motility drug treatment have low efficiency and a high recurrence rate (17). Because current conventional drug treatment mainly addresses the symptoms rather than the pathogenesis, the drugs for treating NERD are constantly updated, but the treatment effect is not satisfactory. A study by Yu et al. (18, 19) shows that the combination of conventional drugs (esomeprazole) with antidepressants or anxiolytics (flupentixol and melitracen) in the treatment of GERD was superior to esomeprazole alone. However, clinical trials have shown that the side effects of even low doses of antidepressants and anti-anxiety drugs limit the widespread use of this combination for the treatment of NERD.

Cognitive-behavioral therapy (CBT) is a psychotherapeutic method that focuses on establishing correct personal cognition through cognitive education and behavioral skills to correct patients’ errors or distortions and to alleviate and eliminate psychological disorders and somatic symptoms. It is quicker and more efficient than other types of psychotherapy (20, 21). As concluded by Glasinovic et al. (22), CBT reduced the number of excessive supragastric belching and improved social and daily activities. The studies published by Kennedy et al. (23) have shown that CBT combined with mebeverine treatment is more effective in patients with irritable bowel syndrome (IBS) than mebeverine treatment alone. Blanchard et al. (24) used CBT to treat patients with IBS. After treatment, their gastrointestinal symptoms and mental status were significantly improved, and this effect persisted over the next 3 months of follow-up. Lackned et al. (25) and others think that CBT can significantly improve gastrointestinal symptoms and relieve anxiety and depression. Therefore, in view of the limitations of conventional NERD drugs and the restriction of the side effects of anti-anxiety drugs and antidepressants, this study seeks to explore the efficacy of non-drug psychotherapy in patients with NERD. Toward this purpose, we established a simple psychological treatment group and psychotherapy combined with conventional drug group for comparison. NERD patients have obvious and long-term unhealed symptoms and experience negative emotions such as anxiety, suspicion, and depression. Patients who believe they have a severe or even incurable condition are especially prone to cognitive anxiety (26–28). Therefore, CBT is theoretically more suitable than other forms of psychotherapy for the treatment of these NERD patients.

However, there are few studies of the therapeutic effects of CBT on NERD, and the mechanism underlying psychotherapy for NERD is not yet clear. In the present work, we explore the correlation between the incidence of NERD and mental and psychological factors and the efficacy of CBT combined with conventional drug treatment of NERD and so deepen the understanding of the pathogenesis of NERD and explore effective treatment.

## Materials and Methods

### Source, Inclusion, and Exclusion Criteria of Cases

A total of 115 patients with NERD who were diagnosed with mood disorder in the Departments of Gastroenterology at Xiangya Hospital and Yiyang Central Hospital from September 2016 to June 2017 were selected. The study was approved by the ethics committees of both hospitals, and all the patients signed the informed consent forms.

#### Inclusion Criteria

(1) Meet the NERD diagnostic criteria: (1) patients with typical heartburn, reflux, and other symptoms lasting for more than 1 month and occurring an average of more than three times per week; (2) patients meeting the previous conditions who also have reflectance disease questionnaire (RDQ) scores over 12 points despite gastroscopy indicating no esophageal mucosal damage (10). (3) Patients whose electron gastroscopy showed no esophageal mucosal damage or Barrett’s esophagus.
(2) Patient’s HAMA >7 points and <21 points, HAMD >7 points and <24 points, or both.

#### Exclusion Criteria

(1) Dysfunction or failure of vital organs; (2) diabetes or neurological disease; (3) contraindications for drugs used in the study; (3) any previous treatment for reflux esophagitis, anxiety, or depression; (5) disinclination to cooperate with the questionnaire or poor medication compliance. Patients were excluded if they met one or more of these conditions.

### Scale Measurement and Observation Indicators

#### RDQ Symptom Questionnaire and Integral Method

The RDQ is currently the most widely recognized and widely applied GERD diagnostic scale in the world, and its validity and reliability in GERD diagnosis have been confirmed (29–31). The RDQ is based principally on the four symptoms: heartburn, reflux (anti-food), non-cardiogenic chest pain, and acid reflux in the respondents over the past 4 weeks. The survey is scored according to the frequency and severity of the survey. Here, 0–5 points with 0 indicating asymptomatic and 5 severe symptoms that affect normal life. All items were added up into the total RDQ score (10, 32, 33).

#### Hamilton Depression Rating Scale (HAMD)

Hamilton Anxiety Rating Scale (HAMA): HAMD and HAMA were used to assess the severity of depression and anxiety, respectively. HAMD contains 17 items yielding a maximum score of 52, with higher scores indicating greater depressive symptom severity. HAMA consists of 14 items.

Hamilton scales are assessed before initiation of treatment and 4, 8, and 12 weeks after initiation of treatment [HAMA score: >7 points and <14 points indicates possible anxiety; >14 points and <21 points indicates definite anxiety; >21 points, certainly have significant anxiety. HAMD score: >7 points and <17 points indicates mild depression; >17 points and <24 points indicates moderate depression; >24 points, indicates severe depression (34–37)].

#### Quality of Life Scale: Using the MOS 36-Item Short-Form Health Survey (SF-36)

SF-36, which has been documented to have acceptable reliability and validity, is used widely to evaluate people’s health-related quality of life. SF-36 is a universal scale that evaluates eight aspects of health-related quality of life: physical functioning, role-physical, bodily pain, general health (GH), vitality (VT), social functioning (SF), RE, and mental health (MH) (38). The eight dimensions use cumulative method to calculate the original scores according to the final question values, and then the original scores are converted to 0–100 conversion scores. Each dimension scores 0 (worst) to 100 (best) (39). SF-36 was administered before treatment and 12 weeks after initiation of treatment.

#### Main Observation Indexes

(1) RDQ symptoms and results of HAMD and HAMA before and after intervention; (2) results of SF-36 before and after intervention.

### Grouping and Therapeutic Regimens

#### Grouping

Patients with NERD who met the criteria were randomly divided into three groups: a drug treatment group, a psychotherapy group, and psychotherapy combined with drug treatment group. There were 12 male and 23 female participants in the drug treatment group (*n* = 35), 14 male and 26 female participants in the psychotherapy group (*n* = 40), and 16 males and 24 female participants in the psychotherapy combined with drug treatment group (*n* = 40). The ages of all subjects were 18–65 years old with a mean age of (46.31 ± 14.45) years old. The tested groups showed no significant differences in gender, age, or occupation.

The patients in the drug treatment group took omeprazole (Trade name: Losec, Sweden AstraZeneca Pharmaceutical Co., Ltd., SFDA approval number: J20080097) 40 mg orally twice a day; domperidone (Xi’an-Janssen Pharmaceutical Co., Ltd. Company, SFDA approval number: H10910084) 10 mg orally every time three times a day for a total of 12 weeks according to doctor’s orders. Patients in the psychotherapy group received CBT alone. Patients in the psychotherapy combined with drug treatment group received the same psychotherapy as in the psychotherapy group and also took conventional drugs.

#### Psychological Intervention Program

In the psychological treatment group and psychotherapy combined with drug treatment group, psychological counselors gave a preliminary diagnosis of psychological problems on the patients and proposed psychological assessment reports. After the establishment of the relationship between the two involved parties, both jointly came to an agreement to formulate a psychotherapy program and perform psychotherapy with CBT twice a week. The details are as follows.

##### Cognitive Adjustment

(1) Clarify the bad cognition regarding the patients’ own symptoms and the distorted perception of themselves and identify automatic thinking; (2) perform the appropriate checks to cultivate an objective basis to help patients rationally analyze their condition, and provide as detailed and patient an explanation as possible of the test results and the prevalence of illness, especially the patients’ suspected symptoms; (3) explain the possible etiology, pathogenesis, treatment, and clinical efficacy of NERD, and psychological factors in the pathogenesis of NERD and cognitive therapy in the relief of NERD symptoms; (4) use authenticity testing and other techniques to help patients alter wrong or distorted cognition and promote cognitive changes.

##### Emotional and Behavioral Adjustments

(1) Introduce emotion and emotional causes and analyze the cycle by which negative emotions and symptoms reinforce each other. Guide patients to use ABC theory to change cognition and improve mood. (2) Master and practice self-discipline training. (3) Guide behavior to help patients develop good eating habits.

During the implementation, the first interview was completed by a professional psychological counselor, and then the patients regularly (twice a week) underwent psychological treatment in the form of small groups to form a treatment alliance, and each patient can benefit from feedback from others and enhance the confidence of treatment. Patients are also required to keep daily records of reflux symptoms, diet, changes in lifestyle, and other matters in the form of homework, which helps identify the interrelationships and determine what the causes of the symptoms are, to allow for appropriate adjustments. In this process, doctors and researchers can also identify patients’ cognitive errors in a timely manner and promptly correct them to ensure that treatment will go smoothly.

##### Efficacy Evaluation Method

Regular follow-up (at 4, 8, and 12 weeks), for scale measurement and RDQ symptom score, and symptoms were recorded and evaluated by the assessors.

###### Efficacy Criteria

The patients’ RDQ scores were used to declare their treatment effective or ineffective after 12 weeks of treatment. Improvement rate

= [(pretreatment score − post treatment score)/pretreatment score] × 100%, improvement rate ≥50% is effective, <50% is ineffective (40).

### Data Processing Methods

We used SPSS 20.0 for data analysis and statistics. HAMD and HAMA of each group before and after treatment were compared and the symptom scores of each group were compared using ANOVA for repeated measurement. The quality of life before treatment and 12 weeks after treatment were compared using paired-samples *T* test. The chi-square test and Fisher exact test were used for the comparison of rate. *P* < 0.05 means that the difference is statistically significant.

## Results

### Comparison of HAMD and HAMA Results

**Figure 1:**
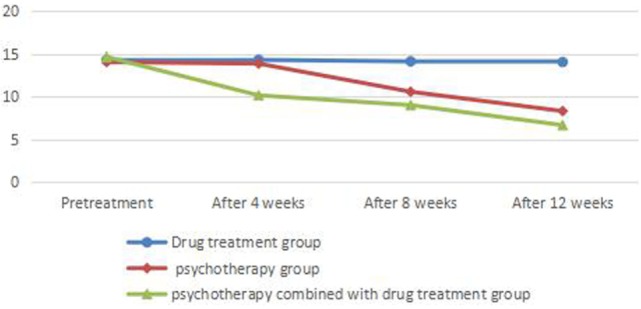
Comparison of HAMD scores before and after treatment in each group.

**Figure 2:**
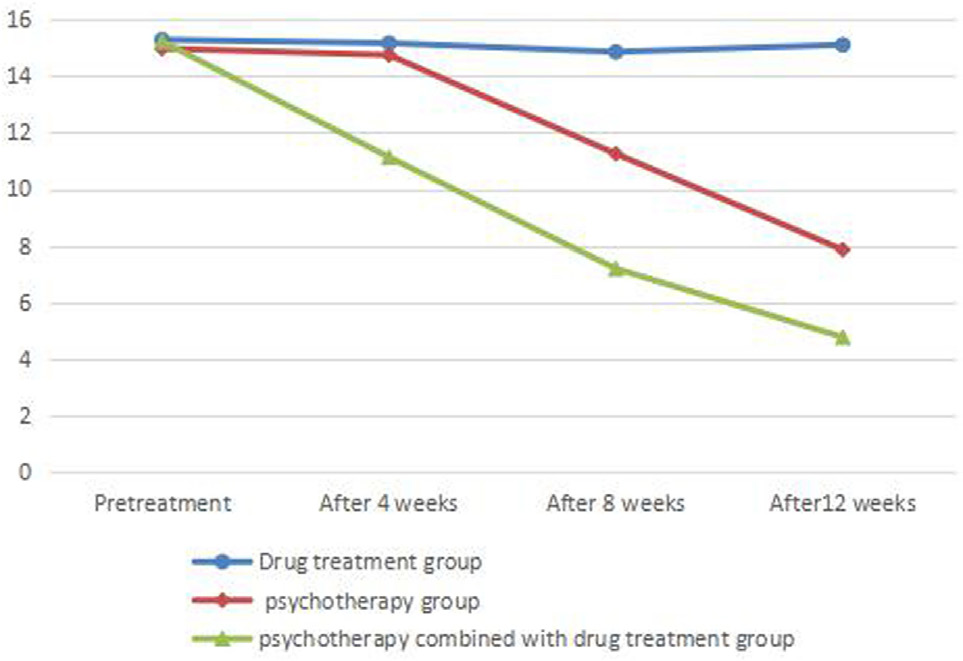
Comparison of HAM scores before and after treatment in each group.

There was no significant difference between HAMD scores before treatment in each group (*P* > 0.05). There was no significant difference in HAMD or HAMA scores before and after treatment in the drug treatment group (*P* > 0.05). There was no significant difference in HAMD or HAMA scores before and at the end of 4 weeks of treatment (*P* > 0.05) and significant difference between HAMD and HAMA scores between before and at the end of 8 and 12 weeks of treatment (*P* < 0.05) in the psychotherapy group. There was a significant difference between HAMD and HAMA scores in the psychotherapy combined with drug treatment group before and at the end of 4, 8, and 12 weeks of treatment (*P* < 0.05) (Tables 1 and 2; Figures 1 and 2).

**Table 1:** Comparison of HAMD scores before and after treatment in each group.

	Drug treatment group	Psychotherapy group	Psychotherapy combined with drug treatment group	*F*	η^2^
Pretreatment	14.26 ± 3.721	14.03 ± 2.948	14.63 ± 4.068	0.281	0.003
After 4 weeks	14.29 ± 3.304	13.85 ± 2.607	10.13 ± 2.747	24.438**	0.180
After 8 weeks	14.11 ± 3.954	10.55 ± 3.419	8.98 ± 2.281	24.052**	0.178
After 12 weeks	14.03 ± 3.981	8.28 ± 3.302	6.63 ± 2.579	51.787**	0.318

*P<0.05.

**P<0.01.

**Table 2:** Comparison of HAMA scores before and after treatment in each group.

	Drug treatment group	Psychotherapy group	Psychotherapy combined with drug treatment group	*F*	η^2^
Pretreatment	15.29 ± 3.383	14.98 ± 3.893	15.23 ± 4.098	0.072	0.001
After 4 weeks	15.17 ± 4.376	14.75 ± 4.634	11.13 ± 3.688	10.660**	0.088
After 8 weeks	14.86 ± 3.942	11.25 ± 3.418	7.18 ± 2.459	50.872**	0.314
After 12 weeks	15.11 ± 4.013	7.85 ± 3.294	4.75 ± 2.048	102.89**	0.481

*P<0.05.

**P<0.01.

### Comparison of NERD Symptom Scores

**Figure 3:**
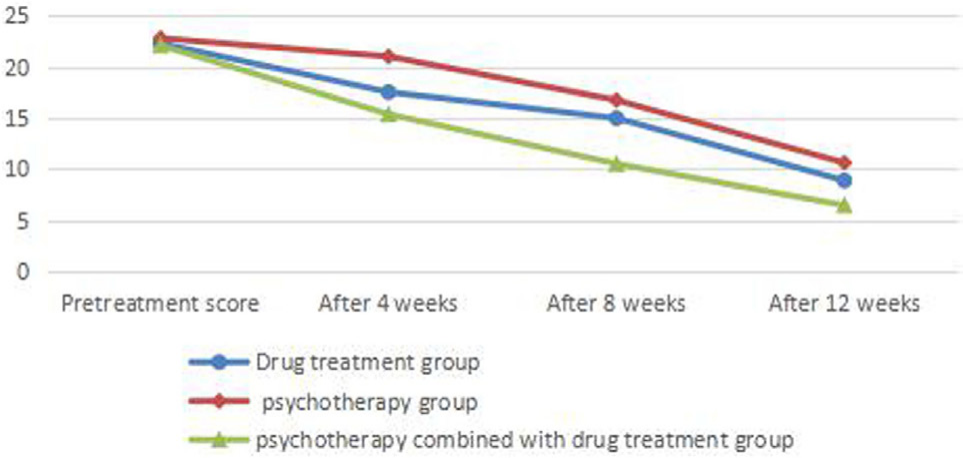
Comparison of symptom scores before and after treatment of patients in each group.

Symptom scores before treatment showed no significant differences between groups (*P* > 0.05). There was significant difference (*P* < 0.05) between before and at the end of 4-, 8-, and 12-week treatment in the drug treatment group; no significant difference between before and at the 4-week treatment (*P* > 0.05) and significant difference between before and at the end of 8 and 12 weeks of treatment (*P* < 0.05) in the psychotherapy group; significant difference between before and at the end of 4, 8, and 12 weeks of treatment (*P* < 0.01) in the psychotherapy combined with drug treatment group. Symptom scores of patients in the psychotherapy combined with drug treatment group were significantly lower than those in other groups at the end of 4, 8, and 12 weeks of treatment (*P* < 0.01) (Table 3; Figure 3).

**Table 3:** Comparison of symptom scores before and after treatment of patients in each group.

	Drug treatment group	Psychotherapy group	Psychotherapy combined with drug treatment group	*F*	η^2^
Pretreatment	22.26 ± 9.516	22.78 ± 9.744	22.08 ± 7.934	0.064	0.001
After 4 weeks	17.51 ± 6.840	21.00 ± 6.013	15.35 ± 5.066	9.083**	0.076
After 8 weeks	14.97 ± 5.732	16.73 ± 6.425	10.50 ± 3.602	14.203**	0.113
After 12 weeks	8.86 ± 3.836	10.60 ± 3.855	6.45 ± 2.521	14.641**	0.117

*P < 0.05.

**P < 0.01.

### Comparison of SF-36

SF-36 scores in NERD patients improved in all categories in the drug treatment group, psychotherapy group, and psychotherapy combined with drug treatment group (Table 4).

**Table 4:** Comparison of SF-36 scores before and after treatment (M ± SD).

	Drug treatment group		Psychotherapy group		Psychotherapy combined with drug treatment group	
			
	Pretreatment	After 12 weeks	Pretreatment	After 12 weeks	Pretreatment	After 12 weeks
Physical functioning	84.03 ± 7.46	87.74 ± 6.31^a^	84.28 ± 7.29	85.63 ± 7.76^b^	83.10 ± 9.06	91.90 ± 6.24^a,b,c^
Role-physical	50.49 ± 7.13	55.63 ± 8.89^a^	50.00 ± 18.15	52.93 ± 15.91	50.83 ± 22.56	63.00 ± 20.01^a,c^
Bodily pain	61.94 ± 8.44	67.00 ± 6.88^a^	61.90 ± 20.44	64.00 ± 21.59	64.68 ± 22.41	77.00 ± 15.97^a,b,c^
General health	70.74 ± 8.12	75.89 ± 8.72^a^	71.60 ± 17.58	79.90 ± 15.76^a^	71.35 ± 19.05	84.10 ± 16.38^a,b^
Vitality	56.14 ± 19.14	62.2 ± 19.22^a^	55.93 ± 20.36	63.10 ± 21.99^a^	55.95 ± 21.72	74.28 ± 18.11^a,b,c^
SF	70.60 ± 20.13	78.6 ± 18.69^a^	70.50 ± 19.18	80.83 ± 14.99^a^	72.85 ± 17.68	87.83 ± 13.48^a,b^
RE	51.97 ± 26.07	57.4 ± 23.95^a^	51.58 ± 20.21	64.03 ± 21.72^a^	50.58 ± 22.44	73.08 ± 21.18^a,b^
Mental health	60.40 ± 15.37	66.7 ± 20.21^a^	60.63 ± 18.68	73.30 ± 12.28^a^	61.08 ± 18.34	76.93 ± 14.05^a,b^

*Compared with prior treatment, ^a^P < 0.05; Compared with the drug group, ^b^P < 0.05; Compared to psychotherapy, ^c^P < 0.05*.

### Comparison of Therapeutic Efficiency

The χ^2^ test showed a significant difference (χ^2^ = 15.53, *P <* 0.01) in symptom improvement between the psychotherapy combined with drug treatment group and the psychotherapy group and another significant difference between the psychotherapy combined with drug treatment group and the drug treatment group (χ^2^ = 6.40, *P* = 0.016). There was no significant difference between the psychotherapy group and the drug treatment group (χ^2^ = 2.32, *P* = 0.05) (Table 5).

**Table 5:** Effects of 12 weeks of treatment in each group.

	Cases	Validity (%)	Invalidity (%)
Drug treatment group	35	26 (74.29)	9 (25.71)
Psychotherapy group	40	23 (57.5)	17 (42.5)
Psychotherapy combined with drug treatment group	40	38 (95)	2 (5)

## Discussion

The results of this study show that the three methods of treatment can all improve patients’ symptoms and quality of life to different degrees. Patients given psychotherapy combined with drug therapy and those given psychological treatment alone showed significantly more pronounced decreases in Hamilton depression, anxiety scale, and RDQ scores after 4, 8, and 12 weeks of treatment than patients who received drug treatment alone. Psychotherapy and drugs in combination showed the best overall curative effect.

The results of this study showed that there was no significant difference in the HAMD and HAMA scores between before initiation of at treatment regimen involving medication alone and after 4, 8, and 12 weeks of such treatment (*P* > 0.05), while the score reflecting RDQ symptoms was significantly improved from the 4th week on, indicating that drug treatment can relieve the symptoms of GERD patients relatively quickly. However, patients often lack a reasonable understanding of their symptoms, and NERD symptoms can easily be misunderstood. The symptoms patients feel are more obvious, which can cause fear and even exacerbate subjective symptoms, so simple drug treatment does not always produce readily visible improvements in the moods of patients and the overall treatment is less effective. We can see that the psychological factors and NERD symptoms interact with each other. In the drug treatment group, there were significant differences in SF-36 scores for quality of life in all dimensions between before and at the end of 12 weeks of treatment, indicating that the alleviation of symptoms helps to improve patients’ quality of life.

There were no significant differences in HAMD, HAMA scores and RDQ symptom scores between before and at the end of 4 week treatment in the psychotherapy group (*P >* 0.05), but significant differences were observed between before and at the end of 8 and 12 weeks of treatment (*P* < 0.05). This suggests that simple psychotherapy can be relatively slow in alleviating mood problems and symptoms. Combined with the comparison of the treatment efficiency, results showed that the effect of psychotherapy on the symptoms is less readily visible than those of psychotherapy combined with drug treatment. We can see that NERD is not a simple psychogenic physical illness, and the cause is rather complicated, so pure psychotherapy cannot completely replace drug treatment. The patients given psychological treatment alone showed significant differences (*P* < 0.05) in VT, GH, emotional function, MH, and social function before and after treatment as indicated using the SF-36 quality of life scale, so this study shows that pure psychotherapy can significantly improve patient quality of life and alleviate the symptoms of NERD, so although psychotherapy cannot completely cure NERD, it is something that most patients need. It further shows that in the course of treatment and rehabilitation of NERD patients, in addition to improving the clinical symptoms of NERD, attention should be paid to the assessment of quality of life.

The cognitive factor is the bridge between the physical and psychological symptoms of NERD patients. Cognitive therapy contributes to the treatment of mental and physical diseases such as NERD by blocking the feedback cycle by which psychological factors and symptoms reinforce and exacerbate each other. Many studies have found that CBT is effective in patients with functional chest pain (41, 42). Drossman et al. (43) compared the efficacy and safety of CBT and a patient education treatment program for adult women with moderate-to-severe functional bowel disease showed the efficacy of CBT to be better than patient education. In this study, the use of CBT as a means of psychological intervention showed a specific effect in improving mood and the quality of life, but hypnotherapy, psychoanalytic therapy, and other treatments are also quite good forms of non-drug psychotherapy (44–46), so it may be best to comprehensively use various psychotherapy tailored to the patient’s personality. In addition, people often pay more attention to the physical illness and neglect the psychological problems of patients in the actual clinical work. The study found that depression to be more prevalent in patients treated by general medical institutions than among those treated in psychiatric hospitals, but physicians’ rate of recognizing mental illness was only 55.6% (47). For this reason, it is necessary to popularize MH knowledge in general hospitals. This is also one of the goals of this study.

The main NERD medications tend to work through acid suppression and improving gastrointestinal motility. They are less effective in treating the mood disorders associated with NERD (48–50). This study showed there to be significant differences in HAMD and HAMA scores between before treatment and after 4, 8, and 12 weeks of treatment in NERD patients with mood disorders in the psychotherapy combined with drug treatment group (*P* < 0.05), and HAMD, HAMA, RDQ, and SF-36 scores of the same period were significantly different from those of the other two groups (*P* < 0.05). This shows that the psychotherapy combined with drug treatment in improving NERD symptoms, relieving depression and anxiety, improving the quality of life and treatment efficiency and other aspects are superior to conventional drug treatment and simple psychotherapy, suggesting that in the treatment of NERD patients with emotional disorders, in addition to conventional PPI and motivation, CBT can improve treatment efficacy.

This may be because CBT promptly improves somatic symptoms, which can relieve the psychological stress and fear associated with the disease, thus alleviating negative emotions such as anxiety and depression (51–53). The study has shown that the patient’s emotional state is closely related to the symptoms of NERD and plays an important role in the progression of NERD (54, 55). Psychological factors can change the secretion of hormones and the movement in the gastrointestinal tract through stress to the brain and gut reflexes. For example, depression and anxiety can regulate esophageal perception, causing the patient to notice low-level esophageal irritations and feel pain, which may be related to the patient’s excessive attention to esophageal reflux events leading to pain-related perception. For these reasons, physicians treating patients with NERD should pay attention to the treatment of mental illness, using psychotherapy to mitigate anxiety and depression, thereby alleviating NERD symptoms and using CBT to ease and eliminate anxiety, depression, other emotional disorders, and somatic symptoms efficiently and persistently (10, 11).

In summary, the etiology of NERD is not yet completely clear. The current studies maintain that it is related to acid reflux, esophageal hypersensitivity, esophageal mechanical stimulation, motility disorders, psychological and social factors, and other factors. They also maintain that the patient’s psychological and spiritual factors also play the role in the pathogenesis of NERD and that they can be why the symptoms persist. Therefore, conventional acid suppression and motility drug treatment combined with psychotherapy for NERD is worth promoting.

This work has some specific limitations: Small sample size and hospital-based samples representing more severe cases limit the generalizability of our findings. In the process of psychological treatment, patients’ individual differences can have a pronounced impact. Some patients experienced rapid results from CBT, and others had much subtler results. CBT may be more suitable for patients with NERD than other forms of psychological therapy, and this must be taken into account when designing a personalized treatment plans. Second, the relationship between NERD and patients’ MH needs to be verified in further studies.

## Ethics Statement

All patients provided their informed written consent. The study was approved by the Ethics Committee on Human Experimentation of Xiangya Hospital and Yiyang Central Hospital and was conducted in accordance with the principles contained in the Declaration of Helsinki for studies in humans.

## Author Contributions

KW, AL, and XL conceived and designed the experiments. YZ, PL, JL, JY, and ZL conducted the experiments and collected data. XL, JY, and FD analyzed the results. XL and FD wrote the main manuscript text. All authors reviewed the manuscript.

## Conflict of Interest Statement

The authors declare that the research was conducted in the absence of any commercial or financial relationships that could be construed as a potential conflict of interest.
